# Co-Developing CARED: A Personalized Dementia Outcome Measure With Caregivers

**DOI:** 10.1093/geroni/igaf122.3562

**Published:** 2025-12-31

**Authors:** Sara Masoud, Ayse Kuspinar, Eunung Na, Dale Dauphinee, Susan Macaulay, Nancy Mayo

**Affiliations:** UT Health San Antonio, San Antonio, Texas, United States; McMaster University, Hamilton, Ontario, Canada; McMaster, Ontario, Ontario, Canada; McGill University, Montreal, Quebec, Canada; Amazing Women Rock, Amazing Women Rock, Ontario, Canada; McGill, Montreal, Quebec, Canada

## Abstract

**Background:**

Outcome measures for dementia often fail to reflect the wide variation in symptoms, behaviors, and functional changes experienced by persons living with dementia and their caregivers. Standardized tools may not capture what matters most to patients and families, limiting clinical relevance and applicability. This poster describes the development of the Caregiver-Reported Outcome Measure for Dementia (CARED), a novel tool co-developed by researchers and family caregivers that integrates both standardization and personalization.

**Methods:**

CARED was developed through a community–academic partnership in which family caregivers served as co-researchers. Caregivers contributed to defining domains, refining items, and making decisions about measure structure. Surveys, co-created with community partners, collected caregiver responses from the US and Canada to identify CARED domains and items.

**Results:**

The final measure consists of 27 total items, four of which are core items to be completed by all respondents, and 23 additional items from which caregivers select the four most relevant to their situation. The personalized structure of CARED ensures the inclusion of universally important outcomes while accommodating individual caregiving circumstances. The co-development process demonstrates the feasibility and value of embedding caregiver partners as partners in measurement design.

**Implications:**

CARED represents an innovative approach to dementia outcomes measurement. Through a partnership of researchers and family caregivers, the tool was developed with the lived experiences and perspectives of the population of interest. This framework may guide future efforts to develop outcome measures that are both personalized and standardized, ensuring greater clinical applicability and responsiveness to patient and caregiver needs.

